# Papillary glioneuronal tumor: a new entity awaiting inclusion in WHO classification

**DOI:** 10.1186/1746-1596-2-6

**Published:** 2007-02-08

**Authors:** BD Radotra, Yashwant Kumar, Alka Bhatia, Sandeep Mohindra

**Affiliations:** 1 Department of Histopathology, Postgraduate Institute of Medical Education and Research, Chandigarh, 160012, India; 2 Department of Neurosurgery, Postgraduate Institute of Medical Education and Research, Chandigarh, 160012, India

## Abstract

Papillary glioneuronal tumor (PGNT) is a recently described lesion of the brain, which is still not included as a separate entity in WHO classification. To date 32 cases of PGNT have been reported in the world literature. We report the 33^rd ^case, a 41-year-old male who presented with pain in the nape of the neck. MRI showed a large, predominantly solid mass involving the cerebral parenchyma of the left temporal and parieto-occipital lobes with extension across the midline. Histologically, it was a mixture of glial and neuronal components. Architecturally, the tumor was notable for its pseudopapillary pattern with hyalinized vessels. PGNT is considered as a low grade neoplasm and surgical excision has been curative in most of the cases. More cases of PGNT need to be reported as they may add further knowledge about its biologic behavior and allow its recognition and classification.

## Background

Tumors of mixed glioneuronal type like gangliocytoma, dysembryoplastic neuroepithelial tumor, ganglioglioma, anaplastic ganglioglioma and central neurocytoma are well recognized in the central nervous system [1]. In the year 1998 a new variant of mixed glioneuronal tumor was described by Komori et al. [2] which is still not included as a separate entity in the WHO classification. It was composed primarily of glioneuronal elements with prominent pseudopapillary structures. This unusual mixed glioneuronal tumor of the central nervous system was called papillary glioneuronal tumor (PGNT). The pseudopapillae are usually composed of hyalinized vessels covered by a single or stratified layer of glial fibrillary acid protein (GFAP) positive astrocytes. The cells forming neuronal elements include neurocytes, ganglioid cells or ganglion cells within the neuropil which are synaptophysin positive. To date 32 cases of PGNT have been reported in the world literature. We report the 33^rd ^case with a review of the literature.

## Case presentation

**Figure 1:**
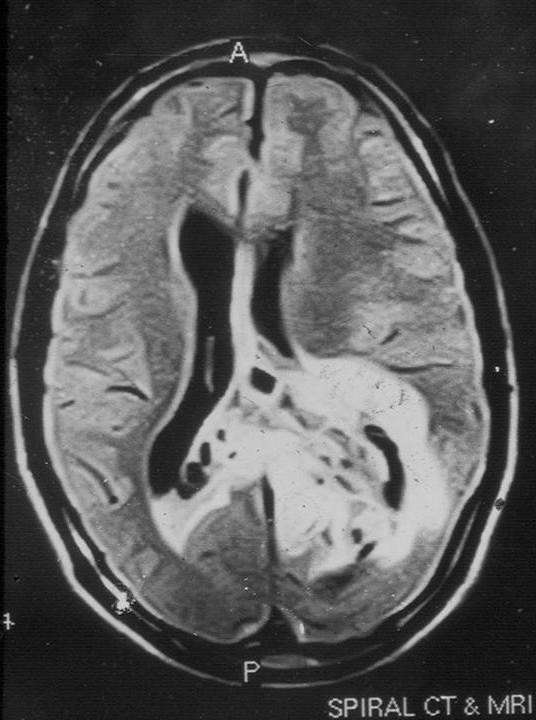
Non-contrast magnetic resonance imaging showing hyper-intense lesion involving the left temporal and parieto-occipital regions. The tumor is crossing the midline to the right parietal region.

The patient was a 41-year-old male who presented with pain in the nape of the neck of one week duration in December 2005. Also there was a history of headache on and off for the past one year and vertigo for two and a half years. Magnetic resonance imaging showed a predominantly solid tumor with ill defined margins. The mass showed a heterogeneous hyper-intense signal on T2 weighted images with no obvious cystic degeneration. It was occupying the cerebral parenchyma of the left temporal and parieto-occipital regions. There was involvement of posterior part of body and splenium of the corpus callosum with extension of the lesion across midline to the right parietal region. The left side of midbrain, thalamus and basal ganglia appeared distorted along with tentorial herniation. The posterior part of the body of the left lateral ventricle was compressed and the third ventricle was displaced to the right side. The tumor was close to but did not reach the cortical surface. Calcification and perilesional edema were absent (Figure 1). Craniotomy was done with near-total excision of the tumor. There were no post operative complications and the patient was discharged after a few days. No radiotherapy was offered to patient and there were no signs of recurrence till last follow up.

## Pathological findings

**Figure 2:**
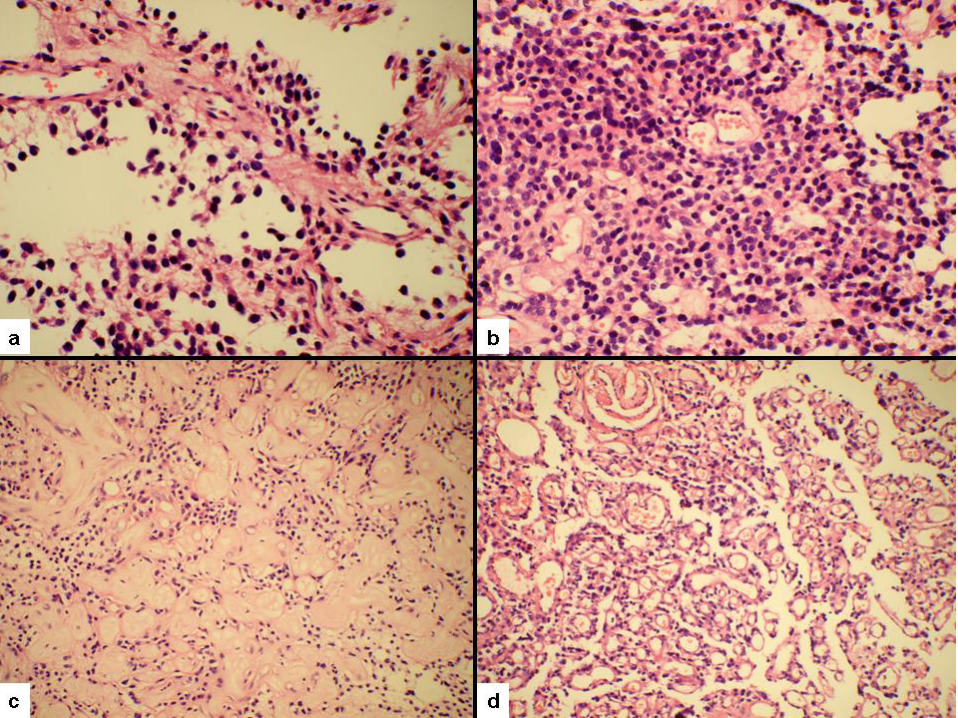
(a) Papillary glioneuronal tumor with pseudopapillary configuration, (b) Solid area of the tumor composed of loosely placed cells with round nuclei and scanty cytoplasm, (c) Marked thickening and hyalinization of the vessels, (d) Angiomatous area with vascular proliferation.

The excised tumor was sent for histological examination in the form of multiple pieces. No capsule or normal looking brain parenchyma could be identified. All the tissue submitted was processed and examined. Microscopically it was a biphasic tumor with pseudopapillary configuration (Figure 2a) and focal solid pattern (Figure 2b). The cores of the pseudopapillae showed thickened vessels lined by single as well as stratified layers of glial cells. In some areas these vessels exhibited marked thickening and hyalinization with obliteration of the lumina (Figure 2c). The solid component of the tumor was formed by sheets of loosely placed cells with round nuclei and scanty cytoplasm. Focal areas formed by oligodendroglial-like, smaller cells having clear cytoplasm were also seen. There was no significant edema, collagen formation or inflammation within the intervening gliofibrillary background.

The presence of angiomatous areas consisting of closely placed smaller and thin walled vessels (Figure 2d) was a unique finding in the present case. These vessels were lined by single layer of endothelial cells with absence of smooth muscle in their wall. These angiomatous areas occupied about one third of the total tumor tissue. In addition a focus of myxoid change was also seen but there were no mature neuronal or ganglionic cells, true or pseudo-rosettes. Mitosis, necrosis and vascular endothelial proliferation were absent.

**Figure 3:**
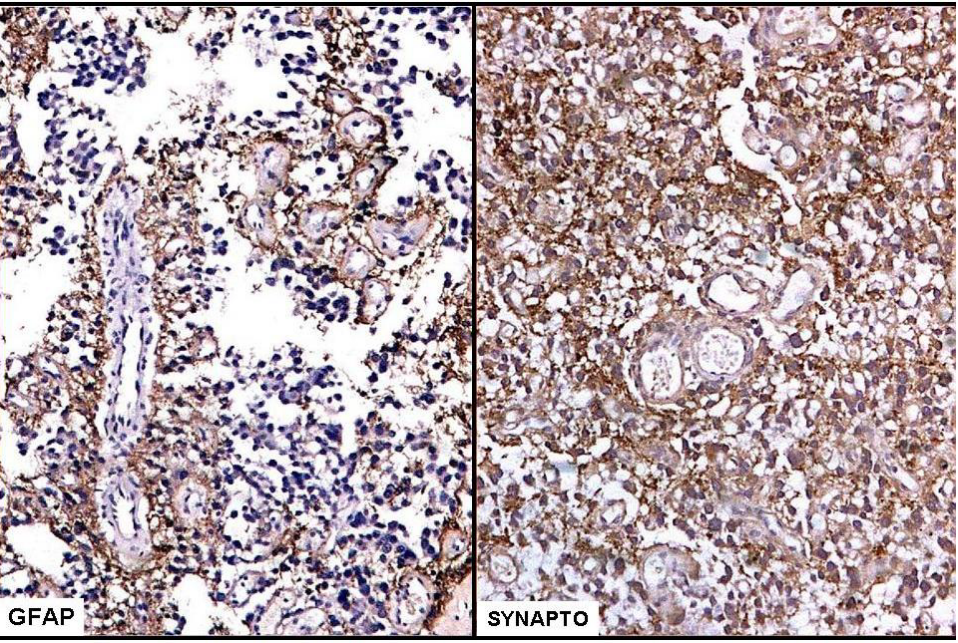
The pseudopapillae formed by glial fibrillary acid protein positive cells and synaptophysin positive neuronal cells forming solid areas. GFAP = glial fibrillary acid protein, Synapto = synaptophysin.

Immunohistochemically most of the glial cells forming pseudopapillae were positive for GFAP and S-100 protein. Neuronal cells forming solid areas were positive for synaptophysin while cells lining the pseudopaillae were negative. Gliofibrillary background in the intervening areas showed GFAP positive staining (Figure 3).

## Discussion

The PGNT is a low grade neoplasm distinct from previously described variants of mixed glioneuronal tumors because of the prominent pseudopapillary architecture and unique clinical and radiographic features. Analysis of all the reported cases in the literature suggests that this tumor has no sex predilection and can affect any age group (range 4–75 years; mean 28 years) [3,4]. Seizures are most common and typify temporal lobe tumors, whereas lesions at other sites usually feature headache and nonspecific signs and symptoms. No focal deficits have been reported so far. Occasionally the patient may be asymptomatic [2,6]. The index case is unusual in the sense that there was no history of seizures despite the temporal location of the tumor. Radiologically it is almost always a cystic lesion, usually with a mural nodule. Size of the tumor may range from 1 cm to 7 cm (mean, 4.6 cm) [2]. The present case differs radiologically from the prior reports in presenting without any obvious cystic change and crossing the midline.

Histologically this tumor shows a biphasic pattern in the form of pseudopapillae and solid areas. The solid areas are mainly composed of neuronal elements which may be in the form of an admixture of small, intermediate or large cells in a fine fibrillary matrix (neuropil). A detailed review of literature suggests that besides these features, degenerative changes are also common in this neoplasm (Table 1). They may be in the form of Rosenthal fibers [2,5,7], calcification, foam cell collection, hemosiderin laden macrophages [2,5,6,8,9] or mild lymphocytic infiltrate [2]. All these degenerative changes were absent in the present case. Minigemistocytic component has been noted in one of the reported cases [9]. Ultrastructurally [2,4] pseudopapillae contain a central vessel surrounded by a duplicated basal lamina, a thick adventitial cuff of collagen and another thin basal lamina separating the surrounding layer of intermediate filament containing astrocytes. The interpapillary region contains neuronal cells. Neurosecretory granules have not been found.

**Table 1:** Pathological features of Papillary glioneuronal tumor

**Pathological features**	**Number (n = 33) **
**Gross features**	

Cystic mass	24
Cystic with mural nodule	10

**Microscopic features**	

Biphasic (glial & neural)	33
Pseudopapillae	33
Hyalinized vessels	28
Rosenthal fibers	13
Microcalcification	8
Pigment laden macrophages	6
Mild lymphocytic infiltrate	5
Minigemistocytes	1
Angiomatous foci	1

**Immunohistochemistry**	

GFAP	33
S-100	31
Synaptophysin	29
Olig2	8/8
NeuN	2/2
MAP2	2/2
NCAM-L	2/2
HNK1	1/1

Differential diagnostic considerations include other neoplasms that may radiologically present as cystic masses with a variably enhancing mural nodule, such as pilocytic astrocytoma and ganglioglioma. The architectural similarities have been noted between PGNT, central neurocytoma and ganglioglioma, however its geographic limitation to the cerebrum suggests a different histogenesis. Other differential diagnoses include ependymoma, extraventricular neurocytic neoplasm and dysembryoplastic neuroepithelial tumor.

Treatment offered to these patients is surgical resection and, if necessary, post operative radio- or chemo-radiotherapy. Follow-up data range from 6 months [2] to 19 years [8] and recurrence has been reported in only one case [9]. All the tumors described had only moderate cellularity. Mitosis, necrosis and endothelial proliferation were not seen in any of the cases. MIB-1 labeling index has also been found to be low (0.5%–2.5%, mean 1.3%) [2,4,7]. The presence of pseudopapillae, degenerative changes and low MIB-1 labeling index all indicate that PGNTs are generally slow growing and low grade neoplasms and thus, appear to carry a good prognosis. Since there has been a rapid increase in the number of reported cases of PGNT, this entity should be included as a separate category in the WHO classification. This would facilitate its wider recognition and help establish an evidence-based approach to treatment.

In conclusion, we report a rare brain tumor, the PGNT in a 41-year-old male with bilateral involvement without any obvious cystic change. On microscopy there was extensive angiomatous change. To the best of our knowledge these changes have not been reported previously.

## Competing interests

The author(s) declare that they have no competing interests.

## Authors' contributions

BDR has made corrections in the manuscript and given approval for publication.

YK has designed, carried out acquisition and analysis of data and drafted the manuscript.

AB has given her valuable suggestions and has helped in drafting of the manuscript.

SM has provided the relevant clinical information.
